# Nail changes secondary to docetaxel chemotherapy : a case report

**DOI:** 10.1186/1752-1947-2-24

**Published:** 2008-01-28

**Authors:** Qamar Ghafoor, Anula Chetiyawardana

**Affiliations:** 1 University Hospital Birmingham, Birmingham, UK

## Abstract

**Introduction:**

Docetaxel is a chemotherapy agent used in the management of many neoplastic conditions. Various side effects are known. Nail changes are often under-recognised or attributed to other causes.

**Case presentation:**

We report the case of a 66 year old gentleman who received docetaxel chemotherapy for non-small cell lung cancer. He had nail changes as a complication of the treatment.

**Conclusion:**

Nail toxicity is a recognised side-effect of taxane chemotherapy agents and can often persist for many months after finishing the treatment. We would like to highlight this problem, so it can be considered as a differential diagnosis in the appropriate population.

## Introduction

Docetaxel is a chemotherapy agent used in the management of many neoplastic conditions. These malignant diseases would include lung, breast, ovary, head and neck and prostate cancer [1,2]. There are various schedules for administering the drug, including weekly and three-weekly. Nail changes are known to happen with all of these schedules.

Docetaxel is a semisynthetic taxane derived from the needles of the European yew (Taxus baccata) [3]. Its mechanism of action is based on binding to tubulin subunits and thus stabilising microtubules. This in turn leads to mitotic arrest and cell death.

Common side-effects related to docetaxel chemotherapy include nausea, peripheral neuropathy, hair loss, neutropenia and oedema. Other complications have been reported including rashes and nail changes.

## Case presentation

A 66 year old gentleman presented to his physician with cough and haemoptysis. He was referred to his local respiratory unit for investigation and found to have non-small cell lung cancer. His TNM staging was IIIB [4] and he was treated according to local guidelines. Initially he had 4 cycles of cisplatin and gemcitabine chemotherapy, followed by external beam radiotherapy to the residual disease and involved lymph nodes. Unfortunately, his disease relapsed and he was given docetaxel as a second line agent.

**Figure 1:**
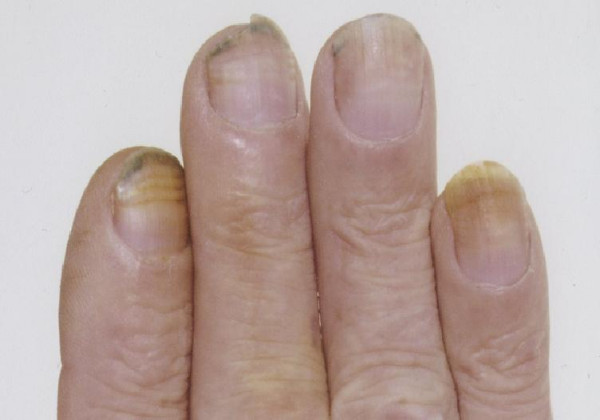
Photograph showing nail changes secondary to docetaxel.

**Figure 2:**
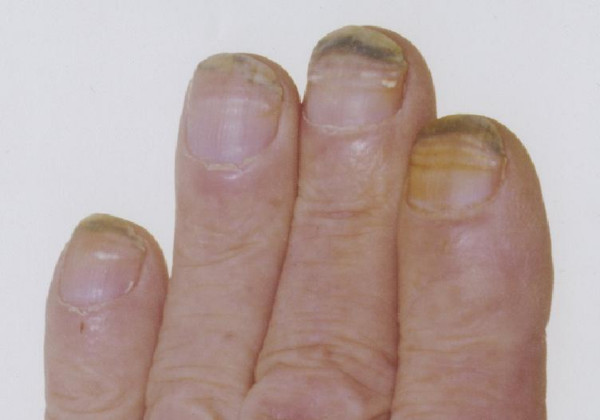
Photograph showing nail changes secondary to docetaxel.

Over the duration of his treatment he began to notice changes in his nails. This involved dyspigmentation of the nail plates in addition to erythema and the formation of nail ridges. (Figures 1 and 2) This was classified as grade 1 nail toxicity using the National Cancer Institute grading system [5].

His general practitioner had been worried about localised infection, but upon assessing the patient we were able to confirm that the nail changes were due to his docetaxel. The decision was made, in agreement with the patient, to persist with his chemotherapy as his disease was showing a response.

Upon completion of his docetaxel his nail changes persisted for months but did improve with time.

## Discussion

Nail changes are a recognised complication of different forms of systemic chemotherapy [5]. Taxane drugs seem to cause more nail toxicity than other drugs. These can include colour changes, beau lines, splinter haemorrhages and onycholysis. The exact mechanisms for these complications are not fully known. There are, however, suggestions that they may be related to changes in the nail matrix in addition to alterations in the vasculature.

There are two grading systems that have been documented in the literature [5]. Firstly that of the National Cancer Institute (version 2.0). This has grade 1 toxicity showing discolouration, ridging or pitting; and grade 2 which has partial or complete loss of nail(s) or pain in the nailbeds. A further classification system has been suggested by Spazzapan S et al [5]. Here grade 1 is discolouration, ridging or pitting. Grade 2 is partial loss of nail, or pain in nailbeds not interfering with function. In grade 3 toxicity there is partial loss of nails or pain in nailbeds interfering with function, or complete loss of nail.

There is no formal protocol to suggest how to deal with the nail toxicity. Often this is done at the clinician's discretion, taking into account a variety of factors. These would include aims of the treatment (curative or palliative), severity of toxicity, other treatment options available and patient choice. Oncologists have been known to manage the toxicity with dose delays or dose reductions, closer observation of the patient, or discontinuation of the taxane agent.

## Conclusion

Nail toxicity is a recognised complication of taxane chemotherapy agents and can often persist for many months after finishing the treatment. Taxanes are used in the management of many malignant diseases. They should be considered in the list of differential diagnosis for nail changes in this population.

## Competing interests

The author(s) declare that they have no competing interests.

## Authors' contributions

QG and AC both managed the case. QG drafted the manuscript and did the literature search. Both authors approved the final manuscript.

## Consent

Written informed consent was obtained from the patient for publication of this case report and any accompanying images. A copy of the written consent is available for review by the Editor-in-Chief of this journal.
